# The Religion of Buddha

**Published:** 1889-06

**Authors:** 


### THE RELIGION OF BUDDHA.

Buddhism is a subject which must continue for a long time to present the student with a boundless field of investigation. No one can bring a proper capacity of mind to such a study, much less write about it clearly, who has not studied the original documents both in Pali and Sanskrit, after a long course of preparation in the study of Vedism, Brahminism, and Hinduism. It is a system which resembles these other forms of Indian religious thought in the great variety of its aspects.

Starting from a very simple proposition, which can only be described as an exaggerated truism—the truism, I mean, that all life involves sorrow, and that all sorrow results from indulging desires which ought to be suppressed—it has branched out into a vast number of complicated and self-contradictory propositions and allegations. Its teaching has become both negative and positive, agnostic and Agnostic.

It passes from apparent atheism and materialism to theism, polytheism and spiritualism. It is under one aspect, mere pessimism ; under another, pure philanthropy ; under another, monastic communism ; under another high morality ; under another, a variety of materialistic philosophy ; under another, simple demonology ; under another, a mere farrago of superstitions, including necromancy, witchcraft, idolatry, and fetichism. In some form or other, it may be held with almost any religion, and embraces something from almost every creed. It is founded on philosophical Brahminism, has much in common with Sankhya and Vedanto ideas, is connected with Vaishnavism, and in some of its phases with both Saivism and Saktism, and yet is, properly speaking, opposed to every one of these systems.

It has in its moral code much common ground with Christianity, and in its mediæval and modern developments, presents examples of forms, ceremonies, litanies, monastic communities, and hierarchical organizations, scarcely distinguishable from those of Roman Catholicism ; and yet a greater contrast than that presented by the essential doctrines of Buddhism and of Christianity can scarcely be imagined. Strangest of all, Buddhism—with no God higher than the perfect man—has no pretentions to be called a religion in the true sense of the word, and it is wholly destitute of the vivifying forces necessary to give vitality to the dry bones of its own morality : and yet it once existed as a real power over at least one-third of the human race, and even at the present moment claims a vast number of adherents in Asia, and not a few sympathizers in Europe and America.

